# Prevalence of Obesity and Related Factors among Bouyei and Han Peoples in Guizhou Province, Southwest China

**DOI:** 10.1371/journal.pone.0129230

**Published:** 2015-06-15

**Authors:** Ke Wang, Dingming Wang, Li Pan, Yangwen Yu, Fen Dong, Ling Li, Li Wang, Tao Liu, Xianjia Zeng, Liangxian Sun, Guangjin Zhu, Kui Feng, Junmei Miao Jonasson, Zhenglai Wu, Ke Xu, Xinglong Pang, Ting Chen, Hui Pan, Jin Ma, Yong Zhong, Bo Ping, Guangliang Shan

**Affiliations:** 1 Institute of Basic Medical Sciences Chinese Academy of Medical Sciences, School of Basic Medicine Peking Union Medical College, Beijing 100005, China; 2 Guizhou Center for Disease Control and Prevention, Guizhou 550004, China; 3 The Section for Social Medicine, Department of Public Health and Community Medicine, Institute of Medicine, The Sahlgrenska Academy at the University of Gothenburg, Gothenburg, Sweden; 4 Peking Union Medical College Hospital, Chinese Academy of Medical Sciences & Peking Union Medical College, Beijing 100730, China; 5 Longli Center for Disease Control and Prevention, Guizhou 551200, China; Shanghai Institute of Hypertension, CHINA

## Abstract

**Objective:**

To investigate the prevalence of general and abdominal obesity and associated factors in Bouyei and Han peoples.

**Design:**

A cross-sectional study was carried out in Guizhou province, southwest China in 2012, with multi-stage sampling to enroll 4551 participants aged 20 to 80 years. General and abdominal obesity were defined by World Health Organization (WHO) for Chinese. A design-based analysis was performed to evaluate prevalence of obesity and its related factors.

**Results:**

Bouyei people had a significantly lower prevalence of general obesity (4.8% vs. 10.9%, *p* < 0.05) and abdominal obesity (13.6% vs. 26.8%, *p* < 0.05) than that in Han people. Prevalence of obesity increased with age until middle-age period and declined thereafter. Men aged 40–49 years group and women aged 50–59 years group have the highest prevalence of general obesity. Prevalence of abdominal obesity was higher than that of general obesity. Middle-age, Higher income, Han people were significantly associated with an increased risk of General/abdominal obesity.

**Conclusions:**

Bouyei people had a lower prevalence of general and abdominal obesity than the Han people. Etiological studies should be conducted to determine underlying genetic factors and dietary factors.

## Introduction

As obesity has become a global epidemic and is closely associated with a number of chronic diseases such as type 2 diabetes, hypertension, coronary artery disease and cancer, it’s now considered as an important public health problem not only in the developed countries but also in the developing countries like China. According to the World Health Organization's definitions of obesity (body mass index, BMI, equal to or more than 30 kg/m^2^), prevalence of obesity was 31% among adults in the United States in 2000 and 21% among men and 23.5% among women in the United Kingdom in 2001[1]. In China, overall prevalence of adult obesity was 2.9% in 2002 (BMI ≥30 kg/m^2^) [2], varying not only by regions, but also by ethnic groups. The prevalence of obesity was significantly lower in southern part of China (1.4% in men and 2.2% in women) than in the north (2.6% in men and 4.9% in women) (BMI (30 kg/m^2^) [3], and the prevalence in western regions of China (9.9%) was lower than that in eastern regions (13.5%) (BMI B28 kg/m^2^) [4]. Many ethnic minorities in southwest China had lower obesity prevalence than Han people [5–7]. Bouyei is one of the ethnic minorities in southwest China, mainly living in Guizhou and Yunnan provinces, with 2.9 million in population. More than 90% of the Bouyei population live in Guizhou province that is situated approximately 24–29° north latitude and 103–109° east longitude. The Guizhou Bouyei people usually live in remote mountainous area at an altitude of 1107 m or higher above sea level. The principal components of their diet are sticky rice, corn, wheat, rice, buckwheat and potatoes. Acid food and cured meat are features of Bouyei diet. Before the 1950s, Bouyei generally didn't intermarry with other ethnic groups and kept primitive lifestyles and their own language. They customarily drank oil-tea camellia. Meat was not generally consumed and it was extremely difficult for the Bouyei people to obtain salt. Since the 1950s, changes have taken place in Bouyei traditional diet and other cultural aspects. A study in 2004 reported that Bouyei had the lowest mean blood pressure among five ethnic populations (Han, Uygur, Kazakh, Tibetan, Bouyei) in China [8]. Some studies have shown hypertension was related to obesity [9,10], so we hypothesize that prevalence of obesity among the Bouyei is different from the Han. At present, there is very limited data regarding current situation of obesity in Bouyei people, and the difference of obesity situation between the Han and the Bouyei in southwest China. The purpose of this study was to investigate prevalence of general and abdominal obesity and its associated factors in Bouyei and Han peoples.

## Materials and Methods

### Selection of Participants

This study is a part of the China National Health survey (CNHS). CNHS is an ongoing nationally representative, and population-based cross-sectional survey conducted in different provinces in China. A cross-sectional study was conducted in Guizhou province, southwest China from October 2012 to December 2012, with multistage sampling to select a representative sample aged 20 to 80 years from general population. In the first-stage, one large city [provincial capital], one developed county, and one underdeveloped county were selected in Bouyei autonomous prefecture. After different cities and counties were chosen, we randomly selected different districts in cities (or county-seats) and rural villages in counties. Then, the sample was stratified according to the sex and age distribution in Guizhou, on the basis of Guizhou’s population data from 2010 (S1 Fig). Participants recruited included residents who had been living in local for more than 1 year. The estimated sample size was 2500 for each race group based on obesity prevalence (12%, according to 2010 China chronic disease monitoring survey [4]) and sampling design (confidence level is 95%; relative error is 15%; design efficiency value is 1.7; no response rate was 10%). A total of 5777 people were selected and invited to participate. 5619 persons completed the questionnaire survey, height and weight measurement. The analysis reported in this article was restricted to the 4551 (response rate was 79%) adults who had the waist circumference measured. Subjects were considered Bouyei or Han people if their parents were both of Bouyei or Han ethnicity. The local government and centers for disease control and prevention (CDC) informed the selected participant before the survey was carried out. They were interviewed at a designated location, such as local CDC or township healthcare centers. The study was approved by the bioethical committee of the Institute of Basic Medical Sciences, the Chinese Academy of Medical Sciences, Beijing, China (approval No. 028–2013). Written informed consent was obtained from all subjects before data collection.

### Questionnaire and physical examinations

All participants were interviewed face-to-face by local CDC staff fluent both in Bouyei language and mandarin to obtain information about demographic characteristics and lifestyle risk factors. Current smokers were defined as those who smoked at least one cigarette per day lasting for at least 6 months, and ex-smokers as those who had stopped smoking more than 6 months prior to the study and stayed abstinent since then. Current drinkers were defined as those who drank at least twice per month with intake more than 640 ml beer or 100 ml Chinese liquor (about 57 g alcohol [11]) lasting for at least 6 months, and ex-drinkers were defined as those who had stopped drinking more than 6 months prior to the study and stayed abstinent since then. Education level was assessed as junior high school or below (less than or equal to 11 years) and senior high school or above (more than or equal to 12 years) by number of years at school with respect to the Chinese schooling system. The average personal monthly income was categorized into 4 groups according to quartiles (less than 250 yuan, 250–999 yuan, 1000–1999 yuan, and not less than 2000 yuan). Occupational physical activity was classed as light for sedentary office workers, shop assistants and general housework, and moderate for trade workers such as carpenters and electrician, and heavy for building and agricultural laborers according to the usual pattern in the past year.

Body height and weight were measured without shoes and in light clothing after overnight fasting. Standing height was measured to the nearest 0.1 cm using a fixed stadiometer and weight was measured to the nearest 0.01 kilometers by BIA (bioelectrical impendence analysis) with a commercially available body composition analyzer (BC-420, TANITA, Japan). Body-mass index (BMI) is a measure of body weight adjusted for body height, which is an important indicator of general obesity in a large population, and it’s calculated as the weight in kilograms divided by the square of the height in meters (kg/m^2^). Considering that abdominal obesity increases the risk for several chronic diseases, we used waist circumference (WC) as an indicator of abdominal obesity, because of its easy measurement and its close correlation with intra-abdominal fat mass confirmed by computed tomographic radiographs [12]. WC was measured at the midway between iliac crest and lowermost margin of the ribs. The mean of three readings of each measurement was taken for statistical analysis. General obesity and abdominal obesity were defined by three criteria as follows: the first criteria is based on WHO suggestions for Chinese [13] (general obesity: BMI ≥27.5 kg/m^2^; abdominal obesity: WC ≥90 cm for men and ≥80 cm for women), the second criteria is recommended by Working Group on Obesity in Chinaq (WGOC) based on the analysis of data collected from 239 972 Chinese adults in the 1990s [14] (general obesity: BMI ≥28 kg/m^2^; abdominal obesity: WC ≥85 cm for men and ≥80 cm for women), and the third one is according to WHO suggestions for Europid [15] (general obesity: BMI ≥30 kg/m^2^; abdominal obesity: WC ≥102 cm for men and ≥88 cm for women)

### Statistical Methods

Statistical analysis was performed using SAS software (version 9.3). Categorical data was described as numbers and percentages. Continuous data were shown as mean plus and minus (±) standard deviation (SD). Between-group differences in subject characteristics were tested using t-test for continuous variables and chi-square/Cochran–Mantel–Haenszel (CMH) test for categorical variables. Sample size was estimated to meet generally recommended requirements for precision in a complex survey design. All calculations were weighted to represent the total population of Han and Bouyei people in Guizhou (20–80 years old) on the basis of Guizhou population data from 2010 and the study sampling scheme, and were corrected for several features of the survey, including oversampling for female, nonresponse, and other demographic or geographic differences between the sample and the total population of Guizhou province. Their variances were estimated by Taylor series linearization [16,17]. In order to compare with national wide data, standardization of prevalence of obesity was performed by direct method adjusted for age and sex structure of the nationwide population according to the 2010 China census data as standard population. The survey-weighted logistic regression model was performed to evaluate association of socio-demographic variables and lifestyle factors with obesity. The *p*-value less than 0.05 was considered to be statistically significant and the significance levels was two-sided.

## Results

### Sociocultural characteristics of two ethnic groups

As shown in Table 1, several variables were significant different between Han and Bouyei people (*p* <0.05). The average age of Han people was younger than that of Bouyei people. Han people had significantly higher height, weight, BMI and WC than Bouyei people. About 70% of the Bouyei had personal income less than 1000 Chinese yuan per month, about 80% of them had education level of junior high school or below (75.6% of the men and 86.3% of the women), and nearly 64% of them engaged in moderate or heavy work (68.6% of the men and 58.6% of the women). About 64.7% of the Han participants came from urban area, while 88.1% of the Bouyei participants came from rural area. Current smokers and alcohol drinker accounted for 28.8% and 37.3% of Han participants, and 34.1% and 44.3% in Bouyei participants, respectively. The prevalence of smoking was higher in men than in women for both ethnic groups, and was higher in Bouyei men than in Han men. The prevalence of alcohol consumption was also higher in men than women in both ethnic group, and was higher in Bouyei men than in Han men, especially the proportion of current drinkers.

**Table 1:** Sociocultural characteristics of the study population: two ethnic groups, Guizhou, southwest China, 2012.

	Overall			Men			Women		
	*Han*	*Bouyei*	*p*	*Han*	*Bouyei*	*p*	*Han*	*Bouyei*	*p*
**NO.**	**2421**	**2130**		**1036**	**1040**		**1385**	**1090**	
**Age** (years)	**48.6±14.1**	**50.5±13.5**	**<.0001**	**49.2±13.9**	**50.6±13.8**	**0.0224**	**48.2±14.3**	**50.5±13.2**	**<.0001**
**Height** (cm)	**157.4±8.3**	**155.2±8.0**	**<.0001**	**164.0±6.4**	**160.8±6.0**	**<.0001**	**152.5±5.9**	**149.8±5.5**	**<.0001**
**Weight** (kg)	**58.3±10.8**	**52.9±9.9**	**<.0001**	**63.9±10.3**	**57.0±9.8**	**<.0001**	**54.1±9.2**	**49.0±8.2**	**<.0001**
**BMI** (kg/m^2^)	**23.5±3.5**	**21.9±3.1**	**<.0001**	**23.7±3.3**	**22.0±3.1**	**<.0001**	**23.3±3.6**	**21.8±3.2**	**<.0001**
**WC** (cm)	**79.4±10.6**	**73.8±9.7**	**<.0001**	**81.8±10.1**	**75.1±9.9**	**<.000**	**77.6±10.5**	**72.6±9.4**	**<.0001**
**Area**			**<.0001**			**<.0001**			**<.0001**
urban	**1565(64.7)**	**254(11.9)**		**679(65.5)**	**110(10.6)**		**886(64.2)**	**144(13.2)**	
rural	**854(35.0)**	**1874(88.1)**		**357(34.5)**	**929(89.4)**		**497(35.9)**	**945(86.8)**	
**Income per month**(RMB yuan)			**<.0001**			**<.0001**			**<.0001**
<250	**352(14.5)**	**694(32.6)**		**188(18.2)**	**510(49.0)**		**333(24.0)**	**663(60.8)**	
250–999	**496(20.5)**	**781(36.7)**		**85(8.2)**	**112(10.8)**		**242(17.5)**	**190(17.4)**	
1000–1999	**560(23.1)**	**211(9.9)**		**200(19.3)**	**128(12.3)**		**360(26.0)**	**83(7.6)**	
≥3(7.	**1013(41.8)**	**444(20.9)**		**563(54.3)**	**290(27.9)**		**450(32.5)**	**154(14.1)**	
**Education level**			**<.0001**			**<.0001**			**<.0001**
Junior high school or below	**1353(56.1)**	**1718(81.1)**		**516(50.1)**	**785(75.6)**		**837(60.6)**	**933(86.3)**	
Senior high school or above	**1059(43.9)**	**401(18.9)**		**515(50.0)**	**253(24.4)**		**544(39.4)**	**148(13.7)**	
**Physical activity**			**<.0001**			**<.0001**			**<.0001**
Light	**1715(71.0)**	**776(36.5)**		**640(61.9)**	**326(31.4)**		**1075(77.7)**	**450(41.4)**	
Moderate or heavy	**702(29.0)**	**1350(63.5)**		**394(38.1)**	**712(68.6)**		**308(22.3)**	**638(58.6)**	
**Smoking status**			**0.0003**			**0.0501**			**0.150**
Never	**1585(65.6)**	**1310(61.5)**		**245(23.7)**	**243(23.4)**		**1340(97.0)**	**1067(97.9)**	
Ex-smoker	**134(5.6)**	**93(4.4)**		**122(11.8)**	**90(8.7)**		**12(0.9)**	**3(0.3)**	
Current	**696(28.8)**	**727(34.1)**		**667(64.5)**	**707(68.0)**		**29(2.1)**	**20(1.8)**	
**Drinking status**			**<.0001**			**<.0001**			**<.0001**
Never	**1424(59.0)**	**1072(50.4)**		**260(25.2)**	**211(20.4)**		**1164(84.4)**	**861(79.0)**	
Ex-drinker	**90(3.7)**	**113(5.3)**		**71(6.9)**	**78(7.5)**		**19(1.4)**	**35(3.2)**	
Current	**899(37.3)**	**942(44.3)**		**702(68.0)**	**748(72.1)**		**197(14.3)**	**194(17.8)**	

Data are expressed as means ± SD or n(%).

BMI, Body Mass Index; WC, waist circumference; *p*, *p* value.

### Sex- and age-specific means of BMI and WC

The relation of mean BMI and mean WC to age was presented in Table 2. In both ethnic groups, mean BMI and WC first increased significantly and then decreased with age, but the trends differed by sex and ethnic group. BMI and WC of men in both ethnic groups peaked at earlier age (40–49 years) than women (60–69 years). The mean BMI in women was relatively lower (Han: 22.7 kg/m^2^ vs. 24.0 kg/m^2^, *p*<0.0001; Bouyei: 21.8 kg/m^2^ vs. 23.0 kg/m^2^, *p* = 0.0001, in 30–39 years) before age of 40 years but higher (Han: 24.2 kg/m^2^ vs. 23.4 kg/m^2^, *p* = 0.0146; Bouyei: 21.4 kg/m^2^ vs. 20.9 kg/m^2^, *p* = 0.0399, in 60–69 years) after age of 59 years than men in the both ethnic groups. Similarly, women had lower mean WC than men before age of 60 years but were not statistically significant different from men in the oldest age groups. Mean BMI and WC of Han people were significantly higher than those of Bouyei people with the same sex across varied age groups (*p*<0.05). Both BMI and WC of the same ethnic and sex group peaked at similar age (Han: 40–49 years in men and 60–69 years in women; Bouyei: 30–39 years in men and 40–49 years in women), while BMI and WC of Bouyei people peaked at much earlier age than Han people with the same sex group.

**Table 2:** Sex- and age-specific means of BMI and WC in Han and Bouyei people.

	Han people			Bouyei people			Men	Women
	*Men*	*Women*	*p-value (Men vs*. *Women)*	*Men*	*Women*	*p-value (Men vs*. *Women)*	*p-value (Han vs*. *Bouyei)*	*p-value (Han vs*. *Bouyei)*
**BMI** (kg/m^2^)								
** 20–29**	**22.3±3.7**	**20.6±2.8**	**0.0002** *	**21.3±3.4**	**20.4±3.0**	**0.1023**	**0.0798**	**0.6302**
** 30–39**	**24.0±3.3**	**22.7±3.5**	**<.0001** *	**23.0±3.1**	**21.8±2.6**	**0.0001** *	**0.0019** *	**0.0029** *
** 40–49**	**24.2±3.4**	**23.6±3.3**	**0.0326** *	**22.9±3.1**	**22.6±3.1**	**0.2557**	**<.0001** *	**0.0002** *
** 50–59**	**24.0±3.2**	**24.1±3.6**	**0.7416**	**22.2±3.0**	**22.1±3.7**	**0.7970**	**<.0001** *	**<.0001** *
** 60–69**	**23.4±3.1**	**24.2±3.8**	**0.0146** *	**20.9±2.4**	**21.4±3.2**	**0.0399** *	**<.0001** *	**<.0001** *
** 70–80**	**23.0±3.2**	**23.5±3.4**	**0.3041**	**20.1±2.4**	**20.3±2.9**	**0.4992**	**<.0001** *	**<.0001** *
**WC**(cm)								
** 20–29**	**77.0±9.9**	**69.9.±8.0**	**<.0001** *	**73.5±9.4**	**69.7±7.9**	**0.0141** *	**0.0242** *	**0.8842**
** 30–39**	**82.8±9.6**	**75.8±10.0**	**<.0001** *	**79.0±9.6**	**72.7±7.3**	**<.0001** *	**0.0002** *	**0.0002** *
** 40–49**	**83.4±10.2**	**77.5±9.0**	**<.0001** *	**77.8±10.1**	**74.7±8.9**	**0.0001** *	**<.0001** *	**0.0001** *
** 50–59**	**82.5±10.0**	**79.8±10.4**	**0.0035** *	**75.7±9.7**	**73.1±10.5**	**0.0067** *	**<.0001** *	**<.0001** *
** 60–69**	**80.8±10.3**	**81.3±11.1**	**0.6692**	**71.3±7.9**	**71.8±10.1**	**0.5340**	**<.0001** *	**<.0001** *
** 70–80**	**80.1±9.7**	**79.8±11.4**	**0.8542**	**68.8±8.1**	**68.9±9.9**	**0.9866**	**<.0001** *	**<.0001** *

Data are expressed as means ± SD.

BMI, Body Mass Index; WC, waist circumference.

* Significantly different between two groups (*p*<0.05).

### Sex- and age-stratified prevalence of obesity

**Fig 1:**
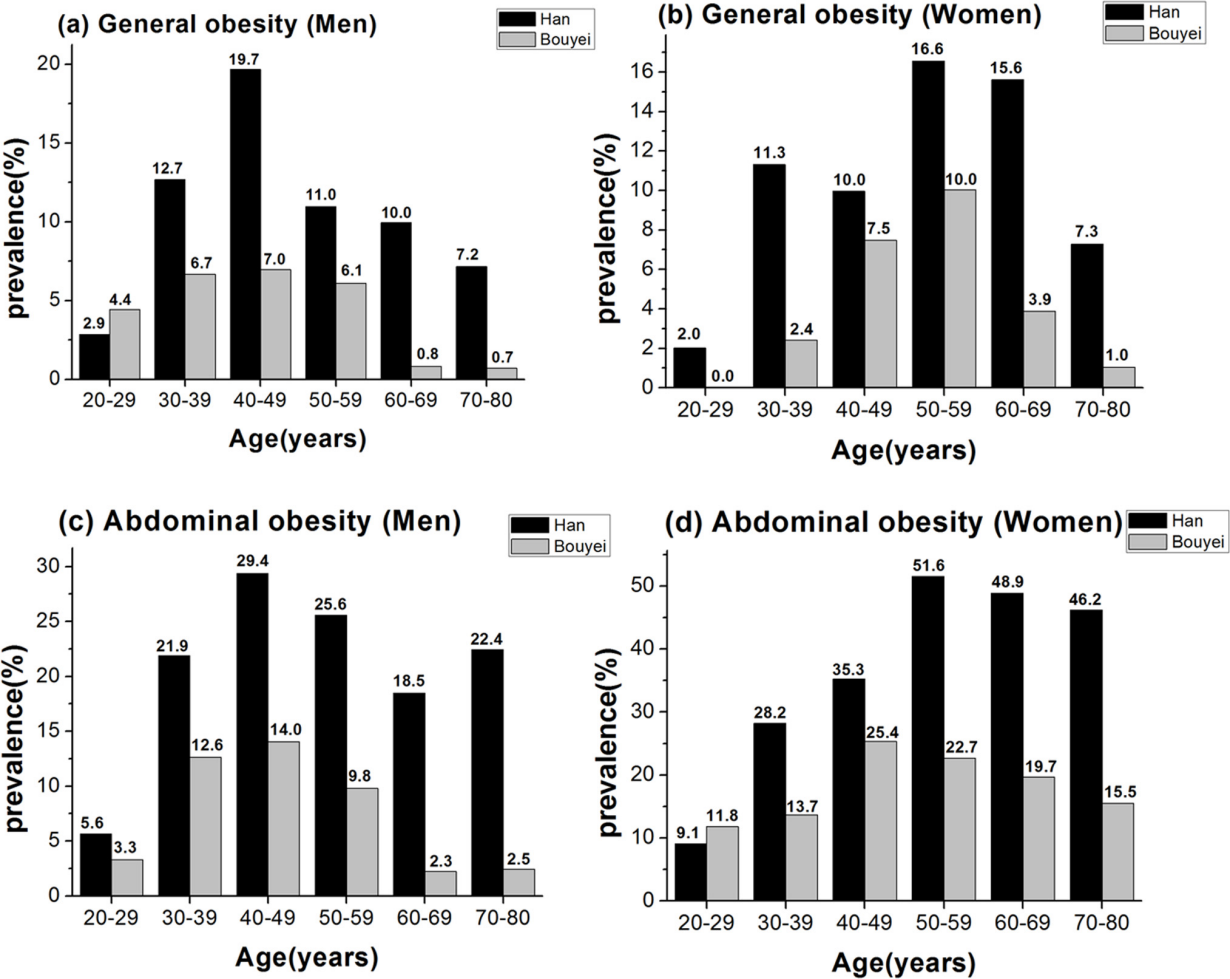
Sex- and age-stratified prevalence of general obesity [(a) and (b)] and abdominal obesity [(c) and (d)] in Han and Bouyei people. General obesity and abdominal obesity were defined by WHO suggestions for Chinese [general obesity: BMI≥27.5 kg/m^2^; abdominal obesity: WC≥90 cm for men and≥80 cm for women].

Fig 1 presents the prevalence of obesity (use the WHO suggestions for Chinese) in different sex and age group of two ethic groups. The prevalence of obesity increased with age at middle-age period and declined thereafter. General obesity prevalence of men peaked at younger age (40–49 years) than that of women (50–59 years) in both ethnic groups. Abdominal obesity prevalence of men also peaked at younger age than that of women in Han people while peaked at similar age between men and women in Bouyei group. General obesity and abdominal obesity prevalence was higher in Han people than in Bouyei people of same sex across different age groups (*p*<0.05), with exception that general obesity prevalence in men (Han vs. Bouyei: 2.9% vs. 4.4%, *p* = 0.4288) and abdominal obesity prevalence (Han vs. Bouyei: 5.6% vs. 3.3%, *p* = 0.4294, in men; 9.1% vs. 11.8, *p* = 0.4079, in women) were not significantly different between two ethic groups at age 20–29.

### Crude and age-standardized prevalence of obesity

**Fig 2:**
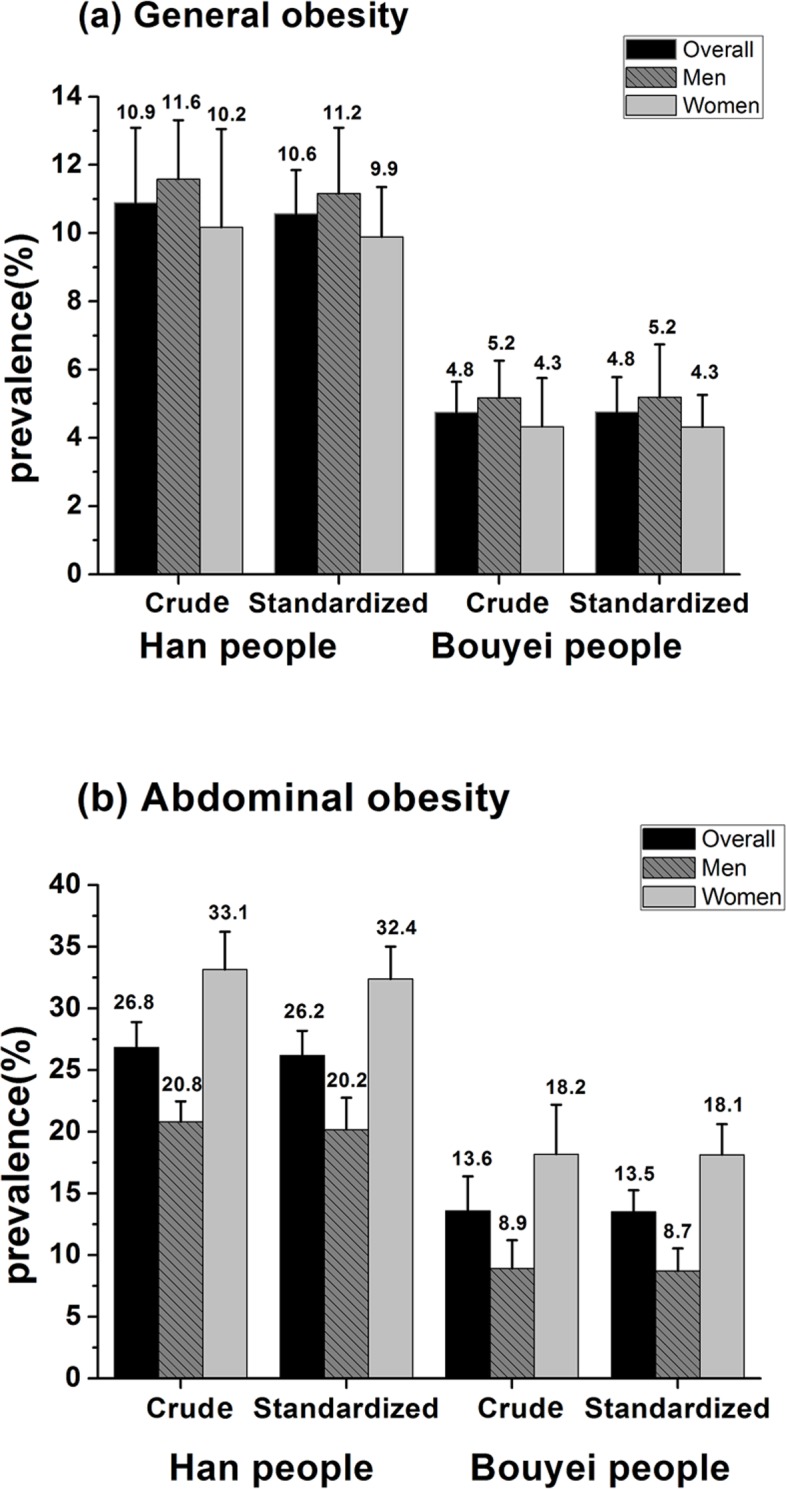
Crude and age-standardized prevalence of general obesity [(a)] and abdominal obesity [(b)] among Han and Bouyei people. Bars indicate 95% confidence intervals. General obesity and abdominal obesity were defined by WHO suggestions for Chinese [general obesity: BMI≥27.5 kg/m^2^; abdominal obesity: WC≥90 cm for men and ≥80 cm for women]. Standardization of prevalence of obesity was adjusted for age structure of the nationwide population according to the 2010 China census data.

After complex weighting, the prevalence of general obesity (Han:10.9% vs. Bouyei:4.8%) and abdominal obesity (Han:26.8% vs. Bouyei:13.6%) was higher in Han people than Bouyei people based on the WHO definition for Chinese (Fig 2). After age-standardization, prevalence of abdominal obesity was higher than that of general obesity. Regardless of obesity types, there was significant difference in prevalence of obesity between the two ethnic groups, with strikingly higher prevalence of obesity in Han people than in Bouyei people (general obesity: Han: 10.6% vs. Bouyei: 4.8%, abdominal obesity: Han: 26.2% vs. Bouyei: 13.5%, *p*<0.001). Men had a slightly higher, however insignificant, prevalence of general obesity than that in women (Han people: 11.2% vs. 9.9%, *p* = 0.1690; Bouyei people: 5.2% vs. 4.3%, *p* = 0.1879). The prevalence of general and abdominal obesity by WGOC criteria for Chinese and by WHO criteria for Europid was provided in supporting information (S1 Table).

### Risk factors for obesity

We used multivariate logistic regression analysis with obesity determined by BMI or WC as dependent variable, respectively. Independent variables included age group, sex, ethnic group, area, personal income per month, education level, physical activity, smoking and drinking status. Middle-aged, higher monthly income, and Han people were significantly associated with an increased risk of general and abdominal obesity. Men had a higher risk for general obesity (OR:1.29, 95% CI: 1.00~1.67) and a lower risk for abdominal obesity (OR:0.40, 95% CI: 0.27~0.60) comparing to women. Compared with light physical activity, people engaged in moderate or heavy labor had lower risk for general obesity (OR:0.79, 95% CI: 0.65~0.98). Ex-smoker had a higher risk for abdominal obesity (OR:1.49, 95% CI: 1.01~2.18) than never-smokers, but not for general obesity. People who lived in urban area had a higher risk for abdominal obesity than those from rural area (OR:1.39, 95% CI: 1.09~1.78).

## Discussion

This study firstly showed that the prevalence of obesity in Bouyei ethnic minority was lower than the majority of Han people in Guizhou of China.

Based on WHO suggested standard for Chinese, the prevalence of obesity among Chinese adults was 10.7% (11.4% in men and 10.1% in women) in 2009 in China Health and Nutrition Survey [18]. Similarly, we also observed 10.9% prevalence in general obesity of Han people (11.6% in men and 10.2% in women) in this study. Based on WHO suggested standard for Europid, the prevalence of obesity was reported 2.6% (2.4% for men and 3.4% for women) according to a China National Nutrition Survey on 221,044 residents aged over 20 years old in 2002 [19]. In our study, prevalence of general obesity was 2.7% in Han people (2.1% for men and 3.4% for women) under similar standard (S1 Table). Based on the criteria of WGOC, the obesity of Han people in Guizhou province was lower than China National obesity prevalence (Han people accounts for 91.6% of the national population). The age-standardized prevalence of general obesity in adults was 12.0% (11.9% for men and 12.1% for women) from 2010 China's chronic disease monitoring [4] and our data demonstrated that age-standardized prevalence of general obesity was 8.8% in Han people (9.7% for men and 8.2% for women) in 2012 (S1 Table). The prevalence of abdominal obesity among Chinese adults, based on WHO suggestions for Chinese, was 37.4% (27.8% in men and 45.9% in women) according to the China Health and Nutrition Surveys in 2009 [18]. This study reported the prevalence of abdominal obesity was 26.8% (men: 20.8% and women: 33.1%) in Han people and was 13.6% (men: 8.9% and women: 18.2%) in Bouyei people (Fig 2), which also can show a trend of lower prevalence of abdominal obesity compared to national prevalence. The cause of this phenomenon may come from demographics distribution and environmental factors. Climate, geography and lifestyle can be different with other region, and economic development level in Guizhou province is among the less developed regions in China. It has been suggested that abdominal obesity is a better risk indicator for the component of the metabolic syndrome, such as hypertension and type 2 diabetes [20,21]. This suggested that the mean value of blood pressure or plasma glucose in Bouyei may be lower than in Han people. It is reported that average blood pressure in southwest area was the lowest in China [22], and Bouyei had the lowest mean blood pressure among five ethnic populations (Han, Uygur, Kazakh, Tibetan, Bouyei) in China [8], these reports were consist with our finding. Because of demographics distribution and investigate time, there are limitations in comparing the obesity prevalence between this study and other studies accurately even under the same obesity definition standard.

**Fig 3:**
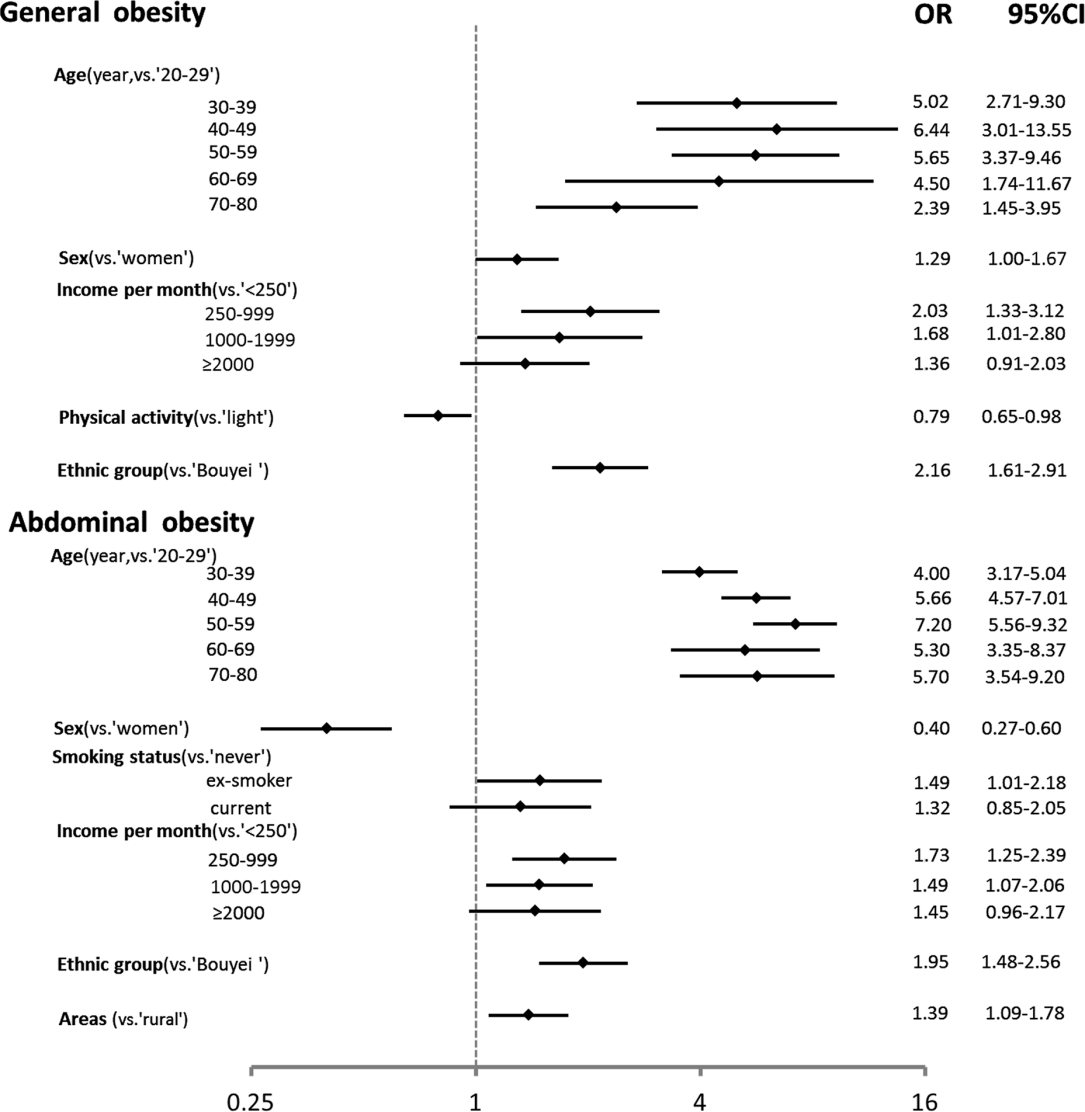
Factors associated with general/abdominal obesity by multivariate logistic regression analysis in Han and Bouyei peoples, Guizhou, southwest China.

This study found that men had a higher risk for developing general obesity than women adjusted for sociodemographic factors (Fig 3). Similarly, general obesity prevalence in Japan is higher in men than women [23]. The 2002 China National Nutrition and Health Survey also reported that men had a slightly higher prevalence of general obesity than women [24]. However, there was no significant difference in prevalence of obesity between men and women participants in Chinese Yi ethnic population [7]. Differences in lifestyle and other factors could explain observed difference in obesity prevalence between sex, countries or regions.

It is important to note that ageing also contributes to higher prevalence of obesity. Consistent with an earlier reports [25,26] and some data based on cross-sectional studies, we have documented that prevalence of general and abdominal obesity prevalence firstly increased and then decreased with age. Interestingly, prevalence of obesity in people over 70 years old was lower than in people of 30~69 years old. It implies that people with longer life span have mild obesity, which may be due partly to obesity-related mortality [27].

Consistent with other studies, our study reported that urban living or life style would make the resident at a higher risk of developing abdominal obesity. Personal income was positively associated with obesity especially abdominal obesity. People in higher income group (250~999 Chinese yuan per month) had 2.03 times higher odds of being general obese and 1.73 times higher odds of being excessive abdominal obese than the lowest income group. This also confirmed our hypotheses that higher income is a risk factor for obesity in a transition society like China. A review in 1989 concluded that high socioeconomic status (SES) showed a consistent positive correlation with obesity prevalence in developing countries [28]. Another review on studies published between 1989 and 2003 showed obesity in the developing world can no longer be considered solely a disease of groups with higher SES and the burden of obesity in each developing country tends to shift towards the groups with lower SES as the country’s gross national product (GNP) increases [29], while our study did not find higher risk of obesity in the group with lower SES. The reason may be that GNP per capita of Guizhou province was the lowest in China from 2001 to 2012, especially the minority habitation in Guizhou, and the current socioeconomic status of minority habitation is similar to Mainland China’s average level in the 1990s.

Some studies reported that ex-smokers were more likely to be obese than never-smokers [30], which was consistent with our study. Other studies reported that cigarette smoking was associated with abdominal and visceral obesity [31,32]. However, our observation didn’t support such finding, and there was a little trend towards a higher prevalence in abdominal obesity with smoking, but not statistical significant. However, it must be considered that the data are cross-sectional only.

Adjusted for sociodemographic factors, prevalence of obesity in Bouyei people was also significantly lower than that in Han people. The age and sex adjusted prevalence of general obesity among Chinese Yi nationality in Sichuan province from 2007 to 2008, southwest China was 7.4% according to WGOC[7]. The age and sex adjusted prevalence of general obesity were 10.5% in Mongolian adults aged 25 years or older[33] and 28.9% and 40.1% in Uygur and Kazakh nationalities aged 35 years or older from 2007 to 2010 in Xinjiang, northwest China, respectively[34].The differences among ethnic groups may come from genetic and lifestyle differences. Further study in association of underlying genetic factors and dietary factors with obesity prevalence is needed. Moreover, Han people deserve more attention in health education for healthier life style. The proportion of Han people associated with obesity risk factors, light physical activity and higher income, was higher than Bouyei people. In developing countries like China, the high income was associated with high-fat diet pattern[35]. With the improvement of living condition and urbanization, Bouyei people may also need to pay attention to the potential risk factors, such as a high-calorie diet and decreased physical activities, even though its current proportion of people associated with the risk factor was significantly lower than Han people.

Potential limitations of the study should be considered. In our study, detailed dietary survey, such as energy intake, was not included in current study. This dietary difference may cause the different prevalence between the two ethnic groups. Even though the diet gap between the Han and Bouyei people become smaller because of cultural exchange, the dietary survey is also needed in further research.

## Conclusions

Bouyei people had a much lower prevalence of general and abdominal obesity than the Han. Further study, such as the association between underlying genetic factors and dietary factors is needed. Prevention strategies between women and men are needed with considering obesity type. Middle-aged men and older women should be given higher priority for obesity preventing and control.

## Supporting Information

S1 Fig (DOCX)

S1 Table (DOCX)
