# Supplementary material for: Prevalence of Obesity and Related Factors among Bouyei and Han Peoples in Guizhou Province, Southwest China
Source: PLoS One. 2015 Jun 15;10(6):e0129230. doi: 10.1371/journal.pone.0129230 (PMC4468129; doi:10.1371/journal.pone.0129230)
Supplement: S1 Fig — (DOCX) [file pone.0129230.s001.docx]

**S1 Fig.**

**Guizhou Province**

**Underdeveloped county**

***Zhenning***

**Developed county**

***Longli***

**Provincial capital**

***Guiyang city***

**2 county-seats**

**Randomly select 12 and 14 rural villages from Longli and Zhenning counties, respectively**

**2 city districts**

**Individual participants**

**8 urban street districts**

**Randomly select 2 urban street districts from each city district or county-seat**

**26 rural villages**

**S1 Fig.** Flowchart of the multistage stratified sampling procedure in the Guizhou province of China National Health Survey.

In the first-stage, the sampling was not random. One large city (provincial capital-Guiyang city, population 445 0,000), one developed county (Longli county) and one underdeveloped county (Zhenning county) were selected. The developed and underdeveloped counties were selected according to degree of economic development status (as assessed on the basis of the gross domestic product [GDP] Per Capita for each county) in Bouyei autonomous prefecture. In the second-stage, a total of two city districts were selected randomly from Guiyang city. In addition, the county-seat was selected from each county. There are total four urban city districts (or county-seats). In the third-stage, two urban street districts were randomly selected from each urban city district (or county-seat). In addition, twelve and fourteen rural villages were randomly selected from Longli and Zhenning counties, respectively. The final stage of sampling was stratified by sex and age distribution based on Guizhou’s population data from 2010.
