# Supplementary material for: Prevalence of Obesity and Related Factors among Bouyei and Han Peoples in Guizhou Province, Southwest China
Source: PLoS One. 2015 Jun 15;10(6):e0129230. doi: 10.1371/journal.pone.0129230 (PMC4468129; doi:10.1371/journal.pone.0129230)
Supplement: S1 Table — (DOCX) [file pone.0129230.s002.docx]

**S1 Table. Crude and age-standardized prevalence of general obesity and abdominal obesity in Han and Bouyei people.**

|  | **Crude prevalence^a^ *% (95%CI)*** | | |  | **Standardized** **prevalence^b^ *% (95%CI)*** | | |
| --- | --- | --- | --- | --- | --- | --- | --- |
|  | *Han people*  *(N=2843)* | *Bouyei people*  *(N=2776)* | *p-value* |  | *Han people*  *(N=2843)* | *Bouyei people*  *(N=2776)* | *p-value* |
| **WHO** |  |  |  |  |  |  |  |
| **BMI**(kg/m^2^) |  |  |  |  |  |  |  |
| Overall | 2.8(2.2- 3.3) | 1.2(0.7- 1.7) | 0.0016^c^ |  | 2.7(2.0-3.3) | 1.2(0.7-1.7) | <0.05^c^ |
| Men | 2.1(1.1-3.2) | 1.1(0.37-1.9) | 0.1296 |  | 2.1(1.2-2.9) | 1.2(0.4-2.0) | >0.05 |
| Women | 3.5(2.9-4.0) | 1.2(0.6-1.9) | <.0001^c^ |  | 3.4(2.5-4.2) | 1.2(0.8-1.7) | <0.05^c^ |
| **WC** (%) |  |  |  |  |  |  |  |
| Overall | 7.6(6.6-8.6) | 2.7(1.9-3.4) | <.0001^c^ |  | 7.4(6.3-8.5) | 2.6(2.0-3.2) | <0.05^c^ |
| Men | 1.7(1.1-2.2) | 0.6(0.3-0.8) | 0.0005^c^ |  | 1.6(0.8-2.4) | 0.5(0.1-1.0) | <0.05^c^ |
| Women | 13.8(11.6-16.1) | 4.7(3.1-6.3) | <.0001^c^ |  | 13.4(11.7-15.1) | 4.6(3.7-5.6) | <0.05^c^ |
| **WGOC** |  |  |  |  |  |  |  |
| **BMI**(kg/m^2^) |  |  |  |  |  |  |  |
| Overall | 8.4(6.5-10.4) | 3.8(3.0-4.6) | <.0001^c^ |  | 8.8 (7.6-10.0) | 4.4 (3.4-5.5) | <0.05^c^ |
| Men | 8.6(7.3-9.9) | 4.1(3.2-5.0) | <.0001^c^ |  | 9.7 (7.8-11.5) | 5.2 (3.5-6.8) | <0.05^c^ |
| Women | 8.2(5.3-11.1) | 3.4(2.4-4.5) | 0.0009^c^ |  | 8.2 (6.9-9.5) | 3.5 (2.7-4.4) | <0.05^c^ |
| **WC** (%) |  |  |  |  |  |  |  |
| Overall | 34.9(33.6-36.2) | 18.0(15.2-20.8) | <.0001^c^ |  | 34.6 (32.3-7.0) | 18.7 (17.7-19.8) | <0.05^c^ |
| Men | 36.6(33.1-40.2) | 17.9(14.7-21.0) | <.0001^c^ |  | 35.4(24.9-31.3) | 18.5(15.7-21.3) | <0.05^c^ |
| Women | 33.1(30.1-36.2) | 18.2(14.2-22.2) | <.0001^c^ |  | 33.7(31.0-36.4) | 19.3(16.7-21.9) | <0.05^c^ |

Abbreviations: WHO, World Health Organisation; WGOC, Working Group on Obesity in China; CI, confidence interval; BMI, Body Mass Index; WC, waist circumference.The criteria recommended by WGOC (general obesity: BMI ≥28 kg/m^2^; abdominal obesity: WC≥85 cm for men and≥80 cm for women) and WHO suggestions for Europid (general obesity: BMI≥30 kg/m^2^; abdominal obesity: WC≥102 cm for men and≥88 cm for women)

***a*** crude prevalence was calculated after complex weighting ; ***b*** Standardized prevalence was calculated after adjusting for age and gender structure of the study population by the 2010 national census; ***c*** Significantly different between two ethnic groups
